# A public–private partnership for facility-based tuberculosis screening using chest radiographs, Viet Nam

**DOI:** 10.2471/BLT.25.293810

**Published:** 2026-05-11

**Authors:** Luan Nguyen Quang Vo, Andrew James Codlin, Tushar Garg, Rachel Forse, Luong Van Dinh, Hoa Binh Nguyen, Tuan Dinh Nguyen, Tom Wingfield, Mohammed Ahmed Yassin, Jamie Tonsing, Minh Huy Pham, Kristi Sidney Annerstedt, Jad Shedrawy, Knut Lönnroth, Jacob Creswell

**Affiliations:** a Friends for International TB Relief, 1/21 Le Van Luong, P. Thanh Xuan, Ha Noi, 100 000, Viet Nam.; b Stop TB Partnership, Geneva, Switzerland.; c National Lung Hospital, Ha Noi, Viet Nam.; d Centre for Tuberculosis Research, Liverpool School of Tropical Medicine, Liverpool, England.; e Global Fund to Fight AIDS, Tuberculosis and Malaria, Geneva, Switzerland.; f United States Agency for International Development, Hanoi, Viet Nam.; g Karolinska Institutet, Department of Global Public Health, WHO Collaboration Centre on Tuberculosis and Social Medicine, Stockholm, Sweden.; h The Swedish Institute for Health Economics, Stockholm, Sweden.

## Abstract

**Objective:**

To determine tuberculosis prevalence among health-care facility patients and to assess the effectiveness of chest radiography in facility-based tuberculosis screening.

**Methods:**

Our cross-sectional analysis used individual-level data collected during 2020–2023 from adults presenting at 796 health-care facilities participating in a public–private partnership in 15 provinces of Viet Nam. We constructed tuberculosis care cascades stratified by symptomatic presentation according to the World Health Organization (WHO) four-symptom screening and chest radiography. We reported diagnostic yield, number needed to screen and positivity.

**Findings:**

Among 1 423 818 participants, 22 598 had bacteriologically confirmed tuberculosis. Diagnostic yields were 2.2% (19 289/892 894) among symptomatic individuals with cough, 1.0% (1869/191 677) among symptomatic individuals without cough and 0.4% (1440/339 247) among asymptomatic individuals. Tuberculosis-suggestive chest radiograph results compared with normal results increased bacteriologically confirmed positivity by 9.4 percentage points in symptomatic individuals with cough (17.3%; 18 746/108 650 versus 7.9%; 543/6851), by 4.6 percentage points in symptomatic individuals without cough (12.6%; 1748/13 831 versus 8.0%; 121/1515) and by 7.6 percentage points in asymptomatic individuals (15.0%; 1328/8862 versus 7.4%; 112/1511). Without chest radiography, the number of individuals requiring testing to detect tuberculosis among symptomatic individuals with cough and without a cough would have been 6.5 (1 084 571/165 679) and 5.4 (892 894/165 679) times higher, respectively.

**Conclusion:**

Chest radiograph screening can reduce the number of expensive molecular tests used in symptomatic patients and enable tuberculosis diagnosis in asymptomatic patients. Future research should analyse cost and cost–effectiveness of using chest radiography in active and intensified case finding.

## Résumé

**Objectif:**

Déterminer la prévalence de la tuberculose chez les patients des établissements de santé et évaluer l’efficacité des radiographies thoraciques dans le dépistage de la tuberculose en milieu hospitalier.

**Méthodes:**

Notre analyse transversale a utilisé des données individuelles recueillies entre 2020 et 2023 auprès d’adultes s’étant présentés dans 796 établissements de santé participant à un partenariat public-privé dans 15 provinces du Vietnam. Nous avons élaboré des cascades de prise en charge de la tuberculose stratifiées en fonction du tableau clinique, selon le dépistage axé sur quatre symptômes de l’Organisation mondiale de la Santé (OMS) et la radiographie thoracique. Nous avons fait état du rendement diagnostique, du nombre de personnes à dépister et du taux de positivité.

**Résultats:**

Sur 1 423 818 participants, 22 598 présentaient une tuberculose confirmée par examen bactériologique. Les rendements diagnostiques étaient de 2,2% (19 289 sur 892 894) chez les personnes symptomatiques présentant une toux, de 1,0% (1 869 sur 191 677) chez les personnes symptomatiques ne présentant pas de toux et de 0,4% (1 440 sur 339 247) chez les personnes asymptomatiques. Les résultats d’une radiographie thoracique évocatrice d’une tuberculose, comparés à des résultats normaux, ont augmenté le taux de positivité confirmée par examen bactériologique de 9,4 points de pourcentage chez les personnes symptomatiques présentant une toux (17,3%; 18 746 sur 108 650 contre 7,9%; 543 sur 6 851), de 4,6 points de pourcentage chez les personnes symptomatiques sans toux (12,6%; 1 748 sur 13 831 contre 8,0%; 121 sur 1 515) et de 7,6 points de pourcentage chez les personnes asymptomatiques (15,0%; 1 328 sur 8 862 contre 7,4%; 112 sur 1 511). Sans radiographie thoracique, le nombre de personnes nécessitant un dépistage de la tuberculose parmi les personnes symptomatiques avec toux et sans toux aurait été respectivement 6,5 fois (1 084 571 sur 165 679) et 5,4 fois (892 894 sur 165 679) plus élevé.

**Conclusion:**

Le dépistage par radiographie thoracique permet de réduire le nombre de tests moléculaires coûteux employés chez les patients symptomatiques et de procéder au diagnostic de la tuberculose chez les patients asymptomatiques. Les recherches futures devraient analyser le coût et la rentabilité de l’application de la radiographie thoracique dans la recherche active et intensifiée des cas.

## Resumen

**Objetivo:**

Determinar la prevalencia de tuberculosis entre pacientes atendidos en centros sanitarios y evaluar la eficacia de la radiografía de tórax en el cribado de tuberculosis en estos centros.

**Métodos:**

El análisis transversal utilizó datos a nivel individual recopilados entre 2020 y 2023 de personas adultas atendidas en 796 centros sanitarios participantes en una asociación público-privada en 15 provincias de Vietnam. Se construyeron cascadas de atención de la tuberculosis estratificadas según la presentación sintomática, de acuerdo con el cribado de cuatro síntomas de la Organización Mundial de la Salud (OMS) y la radiografía de tórax. Se informaron el rendimiento diagnóstico, el número necesario de personas a examinar y la positividad.

**Resultados:**

De los 1 423 818 participantes, 22 598 presentaron tuberculosis confirmada bacteriológicamente. Los rendimientos diagnósticos fueron del 2,2% (19 289/892 894) en personas sintomáticas con tos, del 1,0% (1869/191 677) en personas sintomáticas sin tos y del 0,4% (1440/339 247) en personas asintomáticas. Los resultados de radiografía de tórax sugestivos de tuberculosis, en comparación con resultados normales, aumentaron la positividad confirmada bacteriológicamente en 9,4 puntos porcentuales en personas sintomáticas con tos (17,3%; 18 746/108 650 frente a 7,9%; 543/6851), en 4,6 puntos porcentuales en personas sintomáticas sin tos (12,6%; 1748/13 831 frente a 8,0%; 121/1515) y en 7,6 puntos porcentuales en personas asintomáticas (15,0%; 1328/8862 frente a 7,4%; 112/1511). Sin radiografía de tórax, el número de personas que habrían requerido pruebas para detectar tuberculosis entre personas sintomáticas con tos y sin tos habría sido 6,5 (1 084 571/165 679) y 5,4 (892 894/165 679) veces mayor, respectivamente.

**Conclusión:**

El cribado mediante radiografía de tórax puede reducir el número de pruebas moleculares costosas utilizadas en pacientes sintomáticos y permitir el diagnóstico de tuberculosis en pacientes asintomáticos. Las futuras investigaciones deberían analizar el coste y la eficiencia según los recursos del uso de la radiografía de tórax en la búsqueda activa e intensificada de casos.

## ملخص

**الغرض:**

تحديد مدى انتشار السل بين المرضى المترددين على المرافق الصحية، وتقييم فعالية تصوير الصدر بالأشعة أثناء الكشف عن السل في هذه المرافق.

**الطريقة:**

قمنا في تحليلنا المقطعي باستخدام بيانات على مستوى الأفراد، تم جمعها خلال الفترة من 2020 إلى 2023 من أشخاص بالغين يترددون على 796 مرفقًا صحيًا مشاركًا في شراكة بين القطاعين العام والخاص في 15 مقاطعة في فييت نام. وقمنا بتخطيط مسارات لرعاية مرضى السل، مصنفة حسب الأعراض الظاهرة وفقًا لفحص منظمة الصحة العالمية (WHO) للأعراض الأربعة وتصوير الصدر بالأشعة. قمنا بالإبلاغ عن نتيجة التشخيص، وعدد الحالات التي تحتاج إلى فحص، ونسبة النتائج الإيجابية.

**النتائج:**

من بين 1423818 مشاركًا، تم تأكيد إصابة 22598 منهم بالسل عن طريق الفحص الجرثومي. كانت نتيجة التشخيص %2.2 (19289/892894) بين الأفراد الذين يعانون من أعراض السعال، و%1.0 (1869/191677) بين الأفراد الذين يعانون من أعراض دون سعال، و%0.4 (1440/339247) بين الأفراد الذين لا يعانون من أعراض. أما نتائج تصوير الصدر بالأشعة، والتي تشير إلى الإصابة بالسل، فقد أظهرت مقارنةً بالنتائج الطبيعية زيادة في الإيجابية المؤكدة عن طريق الفحص الجرثومي بنسبة 9.4 نقطة مئوية لدى الأفراد الذين يعانون من أعراض السعال (%17.3؛ 18746/108650 مقابل %7.9؛ 543/6851)، وبنسبة 4.6 نقطة مئوية لدى الأفراد الذين يعانون من أعراض دون سعال (%12.6؛ 1748/13831 مقابل %8.0؛ 121/1515)، وبنسبة 7.6 نقطة مئوية لدى الأفراد الذين لا يعانون من أعراض (%15.0؛ 1328/8862 مقابل %7.4؛ 112/1511). لولا تصوير الصدر بالأشعة، لكان عدد الأفراد الذين يحتاجون إلى فحص للكشف عن السل بين المصابين بأعراض السعال، وبين غير المصابين به، أعلى بمقدار 6.5 (1084571/165679)، و5.4 (892894/165679) مرة على التوالي.

**الاستنتاج:**

يمكن لفحص الصدر بالأشعة أن يقلل من عدد الاختبارات الجزيئية باهظة السعر المستخدمة مع المرضى الذين يعانون من أعراض، وأن يسمح بتشخيص السل لدى المرضى الذين لا تظهر عليهم أعراض. يجب أن تتولى الأبحاث المستقبلية تحليل التكلفة وفعالية التكلفة المتعلقة باستخدام تصوير الصدر بالأشعة في الكشف النشط والمكثف عن الحالات.

## 摘要

**目的:**

旨在确定医疗卫生机构患者中的结核病患病率，并评估胸片检查在基于医疗机构的结核病筛查中的有效性。

**方法:**

我们的横剖分析运用了在 2020 至 2023 年期间从越南 15 个省内参与公私伙伴关系的 796 家医疗卫生机构的成年患者中收集的个体层面数据。我们构建了根据世卫组织 (WHO) 的四症状结核筛查和胸片结果按症状表现分层的结核病护理级联。我们汇报了诊断检出率、需要接受筛查的人数以及阳性率。

**结果:**

在 1423818 名参与者中，有 22598 名患有细菌学确诊结核病。有咳嗽症状的个体、有其他症状但无咳嗽症状的个体以及无症状个体中的诊断检出率分别为 2.2% (19289/892894)、1.0% (1869/191677) 和 0.4% (1440/339247)。与正常胸片结果相比，提示患有结核病的胸片结果致使有咳嗽症状的个体、有其他症状但无咳嗽症状的个体以及无症状个体中的细菌学确诊阳性率分别提高了 9.4 个百分点【17.3% (18746/108650) 对比 7.9% (543/6851)】、4.6 个百分点【12.6% (1748/13831) 对比 8.0% (121/1515)】和 7.6 个百分点【15.0% (1328/8862) 对比 7.4% (112/1511)】。如未采用胸片检查，则有咳嗽症状的个体和有其他症状但无咳嗽症状的个体中需要接受检测方能发现患有结核病的人数将分别高出 6.5 倍 (1084571/165679) 和 5.4 倍 (892894/165679)。

**结论:**

胸片筛查可减少针对有症状患者采用昂贵的分子检测的次数，并可实现针对无症状患者的结核病诊断。未来研究应就针对主动发现病例和强化发现病例采用胸片检查的成本和成本效益展开分析。

## Резюме

**Цель:**

Определить распространенность туберкулеза среди пациентов медицинских учреждений и оценить эффективность рентгенографии органов грудной клетки при скрининге туберкулеза на базе учреждений.

**Методы:**

В рамках данного поперечного исследования использовались индивидуальные данные, собранные в 2020–2023 гг. среди взрослых пациентов, обратившихся в 796 медицинских учреждений, участвующих в государственно-частном партнерстве в 15 провинциях Вьетнама. Авторы построили каскады оказания помощи при туберкулезе, стратифицированные по наличию симптомов в соответствии со скринигом на основе четырех симптомов Всемирной организации здравоохранения (ВОЗ) и результатами рентгенографии органов грудной клетки. Были рассчитаны: диагностическая выявляемость, число обследуемых, необходимое для выявления одного случая, и доля положительных результатов.

**Результаты:**

Среди 1 423 818 участников у 22 598 был бактериологически подтвержденный туберкулез. Диагностическая выявляемость составила 2,2% (19 289/892 894) среди лиц с симптомами в виде кашля, 1,0% (1869/191 677) среди лиц с симптомами без кашля и 0,4% (1440/339 247) среди лиц без симптомов. Результаты рентгенографии органов грудной клетки, указывающие на туберкулез, по сравнению с нормальными результатами увеличивали долю бактериологически подтвержденных случаев на 9,4 процентного пункта у лиц с симптомами в виде кашля (17,3%, 18 746/108 650 по сравнению с 7,9%, 543/6851), на 4,6 процентного пункта у лиц с симптомами без кашля (12,6%, 1748/13 831 по сравнению с 8,0%, 121/1515) и на 7,6 процентного пункта у лиц без симптомов (15,0%, 1328/8862 по сравнению с 7,4%, 112/1511). Без использования рентгенографии органов грудной клетки общее число лиц, которых необходимо протестировать для выявления туберкулеза, среди пациентов с симптомами в виде кашля и без него было бы выше в 6,5 раза (1 084 571/165 679) и 5,4 раза (892 894/165 679) соответственно.

**Вывод:**

Скрининг с использованием рентгенографии органов грудной клетки может сократить число дорогостоящих молекулярных тестов у пациентов с симптомами и обеспечить выявление туберкулеза у лиц без симптомов. В дальнейшем необходимо провести анализ затрат и экономической эффективности применения рентгенографии в рамках активного и усиленного выявления случаев заболевания.

## Introduction

In the early 20th century, chest radiograph was used for mass screening for tuberculosis across Europe and the United States of America, but in 1974 the World Health Organization (WHO) recommended against this practice because it was deemed cost-ineffective and untenable for high-burden countries.^1^ In 2013, WHO released tuberculosis screening guidelines for high-prevalence populations based on evidence from the use of symptom screening and rapid evaluation.^2^^–^^4^ Using symptom screening is fast and inexpensive, and formed part of the original directly observed treatment, short-course (DOTS) strategy that focused on detecting people with smear-positive tuberculosis.^5^

However, according to a review published in 2021, major improvements in radiograph technology (incorporating findings from 26 tuberculosis prevalence surveys) have revealed that chest radiography correctly diagnosed 89% (range: 73–98) of patients with tuberculosis, while symptom screening correctly diagnosed only 50% (range: 36–80), renewing interest in the use of chest radiography.^6^ In 2016, WHO consequently recommended the use of chest radiography to detect tuberculosis in asymptomatic patients.^7^ These recommendations accelerated innovation in safe and portable radiograph technology, as well as unprecedented investment in chest radiography fleet capacity.^8^^,^^9^ Today, chest radiography is a fixture in community-based active case finding, providing cost-effective access to molecular WHO-recommended rapid diagnostics for asymptomatic tuberculosis patients without disease literacy or the economic means to seek health care.^10^^–^^12^

In Viet Nam, community-based active case finding evolved from using mobile radiograph vans to ultra-portable, battery-powered radiograph devices, later scaled up by the national tuberculosis control programme.^8^^,^^13^ Health-care policy implementers observed from these initiatives the high proportion of tuberculosis patients that do not report symptoms and for whom chest radiography is critical in their diagnosis.

However, community-based active case finding using chest radiography is expensive and logistically challenging, and the utility of using chest radiography versus symptoms for facility-based intensified case finding is less apparent than compared with community settings.^14^^,^^15^ Studies of small populations living with human immunodeficiency virus (HIV) have shown that chest radiography is less sensitive than screening for cough.^16^^–^^18^ Conversely, in key HIV-negative groups such as household contacts, children and diabetics, chest radiography offers marginal benefits over the standard WHO four-symptom (i.e. cough, fever, weight loss and night sweats) screening.^19^^–^^21^

In 2020, the Viet Nam National Tuberculosis Control Programme introduced a new first-line diagnostic algorithm for the detection of tuberculosis through its public–private partnership. All patients with tuberculosis-related abnormalities on a chest radiograph received molecular testing for *Mycobacterium tuberculosis*, including detection of rifampicin resistance, using the Xpert® MTB/RIF assay (Cepheid, Sunnyvale, United States of America). This algorithm, locally referred to as Double-X,^22^ was initially piloted for community-based active case finding and subsequently extended to health-care facilities. The algorithm is not based on symptoms, as all individuals are screened with chest radiography and the decision to test is based on chest radiograph findings alone. In alignment with the recent focus on and refined definition of asymptomatic tuberculosis,^23^ this approach has expanded WHO-recommended rapid diagnostic access in the private health sector and available public health-care facilities through the programme.^13^^,^^24^^–^^26^

Modelling studies underpinning WHO recommendations have only used different prevalence levels, and not distinguished between community- and facility-based screening.^27^ Further, the relative efficacy of chest radiography and WHO four-symptom screening at health-care facilities among the general population in a low HIV-burden setting has not been investigated. Our aim was therefore to describe tuberculosis prevalence among patients seeking health care at general and specialist health-care facilities, and to assess the effectiveness of chest radiograph in facility-based tuberculosis screening.

## Methods

### Study design and setting

We conducted a cross-sectional secondary analysis of the results of a public health intervention, part of the Model 5 initiative of the national tuberculosis control programme public–private partnership, that systematically deployed the diagnostic algorithm at participating health-care facilities. We received data acquired from 796 health-care facilities (ranging from mono- and polyclinics to tertiary and quaternary hospitals, providing general and specialized services in public and private sectors) across 15 provinces in the Northern (Ha Giang, Ha Noi, Hai Phong, Thai Nguyen and Thanh Hoa), Central (Binh Dinh, Da Nang, Quang Nam and Thua Thien Hue) and Southern regions (Ba Ria-Vung Tau, Binh Duong, Can Tho, Ho Chi Minh City, Long An and Vinh Long) of Viet Nam. The key prerequisite of eligible health-care facilities was the availability of chest radiography. The national tuberculosis control programme reported 44 693 cases of tuberculosis in these 15 areas in 2022, with just over one third (16 589) of these diagnosed from its public–private partnership. 

### Study population

Our study population comprised adults presenting at relevant health-care facilities between January 2020 and December 2023 (online repository).^28^ Adults typically sought care for various acute and chronic respiratory and systemic conditions, but also preventive and statutory examinations, for example, occupational and emigration-related health screening. All of our study participants had results from both WHO four-symptom screening and a chest radiograph. We did not follow up participants to accurately map their care pathway, including whether a molecular test was a direct consequence of a preceding radiographic finding. To account for this ambiguity in the data set and avoid overestimating the proportion that completed the diagnostic algorithm, our analysis excluded patients with large gaps between verbal (including WHO four-symptom) screening and chest radiograph (≥ 7 days) and molecular testing (≥ 30 days). These thresholds were guided by the national tuberculosis control programme.

### Procedure

As part of the patient screening procedure, health workers interviewed and examined patients to acquire data on age, sex, history of tuberculosis, known exposure to active tuberculosis disease and symptomatic presentation, as assessed by WHO four-symptom screening. The outcome of this initial screening did not influence molecular testing decisions. 

Radiologists then conducted chest radiography for all patients, and asked individuals with chest radiograph abnormalities suggestive of tuberculosis (e.g. upper lobe consolidation or infiltration, cavitary lesions, or hilar or mediastinal lymphadenopathy) to provide a sputum specimen for molecular testing. 

Physicians also recommended molecular testing for some patients with normal chest radiograph results if they exhibited other risk factors, for example, contact with someone with drug-resistant tuberculosis, living with HIV or having been treated for over 1 month in the private sector (with its limited quality assurance for medicines and treatment follow-up). Depending on the availability of testing equipment, health workers either tested sputum specimens on-site or transported them to the nearest laboratory. 

For patients with bacteriologically confirmed tuberculosis by a single molecular test, or bacteriologically unconfirmed tuberculosis with a clinical diagnosis, physicians initiated treatment at the participating health-care facility or referred patients to the nearest tuberculosis treatment centre. Physicians referred patients with resistance to rifampicin to the nearest facility of the national tuberculosis control programme with the technical capacity for programmatic management of drug-resistant tuberculosis.

### Data sources

We obtained data from the national tuberculosis control programme, as well as other facility- and hardware-specific sources. Programme sources included a tuberculosis screening application and the routine electronic surveillance system. Facility-specific systems encompassed bespoke patient management and hospital information systems, or patient registers and files in the absence of digital systems. Hardware-specific sources from radiograph and GeneXpert systems provided molecular and chest radiograph results compiled in Excel (Microsoft, Redmond, USA) or as raw extracts from associated equipment.

### Outcomes

Our primary outcomes were diagnostic yield, defined as the proportion of patients diagnosed with tuberculosis among those screened using WHO four-symptom screening and chest radiography, and the number needed to screen per tuberculosis patient detected, defined as the inverse of diagnostic yield. We stratified outcomes by tuberculosis-related symptoms (symptomatic with current cough irrespective of other symptoms, symptomatic without a cough but at least one other symptom, and asymptomatic) and by chest radiograph abnormalities. 

### Statistical analyses

We constructed tuberculosis cascades from verbal assessment to treatment linkage in aggregate, stratified by the three symptom cohorts and chest radiograph results. To assess the diagnostic testing efficiency, we also calculated cohort- and radiograph-stratified positivity, that is, the theoretical proportion of all bacteriologically confirmed patients with tuberculosis in the sample detected with only chest radiograph compared with those detected with only WHO four-symptom screening. 

We considered two-tailed hypothesis tests with a *P*-value of less than 0.05 to be statistically significant. We performed all statistical analyses using Stata version 17 (Statacorp LLC, College Station, USA).

### Ethical considerations

Our study received ethical approval from the Ha Noi University of Public Health Ethics Committee for Biomedical Research (374/2023/YTCC-HD3). The Viet Nam Ministry of Health (3688/QD-UBND) and Ho Chi Minh City People’s Committee (2681/QD-UBND) approved the implementation of the intervention and associated data collection. We conducted all activities in accordance with the Viet Nam national guidelines on the diagnosis, treatment and prevention of tuberculosis (1314/QD-BYT). We pseudonymized all data before analysis.

## Results

### Participant characteristics

Our study sample included 1 423 818 individuals who had undergone WHO four-symptom screening and chest radiography, with a median age of 36 years (interquartile range: 36–56) and 50.2% (714 769) were male (Table 1). We observed that 62.7% (892 894) were symptomatic with cough, 13.5% (191 677) were symptomatic without cough and 23.8% were asymptomatic (339 247). We noted that slightly more men than women screened positively from WHO four-symptom screening (50.8%; 453 703/892 894 with cough; 51.5%; 98 694/191 677 without cough), and that a larger proportion of women were asymptomatic (52.1%; 176 875/339 247). Most (55.1%; 785 010/1 423 818) reported one symptom, and only 0.3% (4 455/1 423 818) reported all four symptoms. About 0.7% (10 290/1 423 818) reported a previous history of tuberculosis or exposure to someone with active tuberculosis disease. Further information on tuberculosis diagnosis in the study population is available in the online repository.^28^

**Table 1:** Sociodemographic and clinical characteristics of participants included in study on utility of chest radiograph for tuberculosis diagnosis, Viet Nam, between January 2020 and December 2023

Sociodemographic and clinical characteristics	Number (%)			
	Total study population (*n* = 1 423 818)	Symptomatic with cough (*n* = 892 894)	Symptomatic without cough (*n* = 191 677)	Asymptomatic (*n* = 339 247)
**Sex**				
Male	714 769 (50.2)	453 703 (50.8)	98 694 (51.5)	162 372 (47.9)
Female	709 049 (49.8)	439 191 (49.2)	92 983 (48.5)	176 875 (52.1)
**Age, years^a^**				
≤ 15	47 446 (3.3)	33 557 (3.8)	7 026 (3.7)	6 863 (2.0)
16–35	307 568 (21.6)	188 591 (21.1)	47 061 (24.6)	71 916 (21.2)
36–55	463 448 (32.6)	286 036 (32.0)	63 235 (33.0)	114 177 (33.7)
> 55	604 979 (42.5)	384 486 (43.1)	74 300 (38.8)	146 193 (43.1)
Missing	377 (NA)	224 (NA)	55 (NA)	98 (NA)
**Region**				
North	431 796 (30.3)	291 562 (32.7)	63 797 (33.3)	76 437 (22.5)
Central	213 961 (15.0)	128 812 (14.4)	28 867 (15.1)	56 282 (16.6)
South	778 061 (54.6)	472 520 (52.9)	99 013 (51.7)	206 528 (60.9)
**Initial screening site^b^**				
Pharmacy	4 046 (0.3)	2 686 (0.3)	755 (0.4)	605 (0.2)
Mono- or polyclinic	299 768 (21.5)	177 788 (20.2)	45 367 (23.8)	76 613 (23.6)
Hospital	1 089 605 (78.0)	699 335 (79.3)	144 002 (75.7)	246 268 (75.7)
Other	3 625 (0.3)	1 838 (0.2)	140 (< 0.1)	1 647 (0.5)
Missing	26 774 (NA)	11 247 (NA)	1 413 (NA)	14 114 (NA)
**No. symptoms from the WHO four-symptom screen**				
Asymptomatic	339 247 (23.8)	0 (0.0)	0 (0.0)	339 247 (100.0)
One symptom	785 010 (55.1)	650 142 (72.8)	134 868 (70.4)	0 (0.0)
Two symptoms	257 053 (18.1)	203 606 (22.8)	53 447 (27.9)	0 (0.0)
Three symptoms	38 053 (2.7)	34 691 (3.9)	3 362 (1.8)	0 (0.0)
Four symptoms	4 455 (0.3)	4 455 (0.5)	0 (0.0)	0 (0.0)
**Cough**				
No	530 924 (37.3)	0 (0.0)	191 677 (100.0)	339 247 (100.0)
Yes	892 894 (62.7)	892 894 (100.0)	0 (0.0)	0 (0.0)
**Fever**				
No	1 152 404 (80.9)	725 310 (81.2)	87 847 (45.8)	339 247 (100.0)
Yes	271 414 (19.1)	167 584 (18.8)	103 830 (54.2)	0 (0.0)
**Night sweats**				
No	1 297 758 (91.2)	841 297 (94.2)	117 214 (61.2)	339 247 (100.0)
Yes	126 060 (8.9)	51 597 (5.8)	74 463 (38.8)	0 (0.0)
**Weight loss**				
No	1 283 091 (90.1)	825 722 (92.5)	118 122 (61.6)	339 247 (100.0)
Yes	140 727 (9.9)	67 172 (7.5)	73 555 (38.4)	0 (0.0)
**History of tuberculosis**				
No	1 413 528 (99.3)	886 881 (99.3)	190 176 (99.2)	336 471 (99.2)
Yes	10 290 (0.7)	6 013 (0.7)	1 501 (0.8)	2 776 (0.8)
**Known exposure to tuberculosis**				
No	1 414 074 (99.3)	888 893 (99.6)	189 533 (98.9)	335 648 (98.9)
Yes	9 744 (0.7)	4 001 (0.4)	2 144 (1.1)	3 599 (1.1)
**Chest radiograph result**				
No signs of tuberculosis^c^	1 258 139 (88.4)	759 155 (85.0)	174 109 (90.8)	324 875 (95.8)
Consistent with tuberculosis^d^	165 679 (11.6)	133 739 (15.0)	17 568 (9.2)	14 372 (4.2)

NA: not applicable.

^a^ Median age of total study population and all cohorts by symptom(s): 36 years.

^b^ First point of contact in the health system and first entry in the data system.

^c^ Includes patients with chest X-rays classified as abnormal, but not tuberculosis.

^d^ Patients with chest radiograph abnormalities suggestive of tuberculosis.

Note: inconsistencies arise in some values due to rounding.

### Tuberculosis screening cascade

Of the total sample screened by chest radiography, we noted that 11.6% (165 679/1 423 818) had abnormalities suggestive of tuberculosis (Table 1). Of these patients, 87.7% (141 220/165 679) received molecular testing (Table 2). Among tested individuals, 22 598 had bacteriologically confirmed tuberculosis, giving a diagnostic yield of 1.6% (22 598/1 423 818), a number needed to screen of 63 (1 423 818/22 598) and a positivity of 16.0% (22 598/141 220), irrespective of WHO four-symptom screening and chest radiograph results. When including bacteriologically unconfirmed tuberculosis, that is, a total of 29 904 patients diagnosed with all forms of tuberculosis (that is, the combination of bacteriologically confirmed and clinically diagnosed active tuberculosis disease), we obtained a diagnostic yield of 2.1% (29 904/1 423 818) and a number needed to screen of 48 (1 423 818/29 904). Of the patients diagnosed with all forms of tuberculosis, 94.3% (28 213/29 904) had chest radiograph results suggestive of tuberculosis. Overall, 90.7% (27 110/29 904) of patients with all forms of tuberculosis began medical treatment.

**Table 2:** Care cascade for participants included in study on utility of chest radiograph for tuberculosis diagnosis, Viet Nam, between January 2020 and December 2023

Care cascade	Total study population (*n* = 1 423 818)			WHO four-symptom screen							
				Symptomatic with cough (*n* = 892 894)			Symptomatic without cough (*n* = 191 677)			Asymptomatic (*n* = 339 247)	
	Consistent with tuberculosis^a^	No signs of tuberculosis^b^		Consistent with tuberculosis^a^	No signs of tuberculosis^b^		Consistent with tuberculosis^a^	No signs of tuberculosis^b^		Consistent with tuberculosis^a^	No signs of tuberculosis^b^
No. assessed by chest radiograph (%)	165 679 (11.6)	1 258 139 (88.4)		133 739 (15.0)	759 155 (85.0)		17 568 (9.2)	174 109 (90.8)		14 372 (4.2)	324 875 (95.8)
No. assessed by chest radiograph with molecular test results (%)	131 343 (79.3)	9 877 (0.8)		108 650 (81.2)	6 851 (0.9)		13 831 (78.7)	1 515 (0.9)		8 862 (61.7)	1 511 (0.5)
No. patients with biologically confirmed tuberculosis (%)	21 822 (16.6)	776 (7.9)		18 746 (17.3)	543 (7.9)		1 748 (12.6)	121 (8.0)		1 328 (15.0)	112 (7.4)
No. patients with all forms of tuberculosis, including clinically confirmed (number needed to screen)^c^	28 213 (50)	1 691 (842)		23 989 (37)	1 086 (822)		2 446 (78)	381 (503)		1 778 (191)	224 (1 514)
No. patients with all forms of tuberculosis linked to care (%)	25 545 (90.5)	1 565 (92.6)		21 695 (90.4)	995 (91.6)		2 278 (93.1)	363 (95.3)		1 572 (88.4)	207 (92.4)

WHO: World Health Organization.

^a^ Patients with chest radiograph abnormalities suggestive of tuberculosis.

^b^ Includes patients with chest X-rays classified as abnormal, but not tuberculosis.

^c^ All forms include both bacteriologically confirmed and clinically diagnosed active tuberculosis disease.

Note: each percentage uses as its denominator the number in the row above.

### Symptomatic versus asymptomatic positivity 

We observed that the symptomatic with cough cohort had the highest proportion of tuberculosis-suggestive chest radiograph results of 15.0% (133 739/892 894) compared with 9.2% (17 568/191 677) in the symptomatic without cough cohort and 4.2% (14 372/339 247) in the asymptomatic cohort (Table 2). The symptomatic with cough cohort also had the highest overall prevalence of bacteriologically confirmed tuberculosis of 2.2% (19 289/892 894; number needed to screen 46), followed by for the symptomatic without cough cohort (1.0%; 1 869/191 677; 103) and the asymptomatic cohort (0.4%; (1 440/339 247; 236). 

Stratifying case detection by WHO four-symptom screening and chest radiograph results (Table 2) shows that chest radiography increased bacteriologically confirmed tuberculosis positivity. In the cohort of symptomatic individuals with cough, positivity was 17.3% (18 746/108 650) in those with chest radiograph results suggestive of tuberculosis-related abnormalities versus 7.9% (543/6851) in those tested despite a normal chest radiograph, which is an increase of 9.4 percentage points. In the cohort with symptomatic individuals without cough, this increase was 4.6 percentage points (12.6%; 1748/13 831 versus 8.0%; 121/1515) and in the asymptomatic cohorts 7.6 percentage points (15.0%; 1328/8862 versus 7.4%; 112/1511). 

Overall, 79.3% (131 343/165 679) of participants with presumptive tuberculosis by chest radiography were tested according to the diagnostic algorithm, compared with 0.8% (9 877/1 258 139) molecular testing in participants with normal chest radiograph images but based on other eligibility criteria (Table 2). Of note, among participants with presumptive tuberculosis by chest radiography, a smaller proportion of the asymptomatic group (61.7%; 8 862/14 372) compared with the symptomatic with cough (81.2%; 108 650/133 739) and without cough (78.7%; 13 831/17 568) cohorts underwent molecular testing (Table 2). 

### Chest radiograph utility 

Of all participants with bacteriologically confirmed tuberculosis, we observed that 96.6% (21 822/22 598) had a chest radiograph suggestive of tuberculosis across the three cohorts (Table 2). In comparison, 93.6% (21 158/22 598) screened positive according to WHO four-symptom screening, and 85.4% (19 289/22 598) screened positive with cough as a symptom. Although the WHO four-symptom and cough screening detected 27 902 and 25 075 participants with all forms of tuberculosis, respectively, the number of individuals who would have required testing to detect tuberculosis in the absence of chest radiography would have been 6.5 (1 084 571/165 679) and 5.4 (892 894/165 679) times higher, respectively, compared with the 165 679 participants identified with abnormal chest radiographs who were subsequently referred to molecular testing. 

## Discussion

For decades, traditional symptom screening for prolonged cough comprised the bedrock of tuberculosis case finding at health-care facilities in settings of high tuberculosis burden, because chest radiography was expensive and not always readily available. In contrast, diagnosis by smear microscopy was inexpensive and could identify patients with high bacterial load, who were likely more infectious relative to tuberculosis-positive patients without a cough.^29^^,^^30^ Although community-based screening with chest radiograph has found higher numbers of tuberculosis cases than from symptom screening because of the detection of asymptomatic tuberculosis, few facility-based studies have been documented.^13^^,^^31^^–^^33^ Active case finding tends to screen more people who self-perceive as healthy and may rather be subject to demographic, social, occupational or behavioural vulnerabilities. Our findings are concordant with a recent study from Cameroon, where chest radiograph identified 10% (3/30) of patients with asymptomatic culture-positive tuberculosis.^18^

Our diagnostic yield among asymptomatic individuals was negatively affected by the lower proportion of molecular testing in asymptomatic patients with tuberculosis-related abnormalities on chest radiograph compared with symptomatic participants; this could be addressed by the development of tests that can process oral swabs.^34^ In a meta-analysis of prevalence surveys only 17.8% (107 229/602 863) of participants reported a cough, compared with almost two thirds in our facility-based screening.^35^ Nevertheless, the utility of chest radiograph in facility-based screening remains high because the lower diagnostic yield compared with community-based screening is offset by the large number of people seeking health care, including those deemed asymptomatic by the WHO four-symptom screening, translating to a larger number of tuberculosis diagnoses.

No screening tool has perfect sensitivity; the reporting of symptoms can be subjective and personality dependent, highlighting the challenges of clearly defining an asymptomatic individual in routine care.^36^^,^^37^ In our study, a small proportion of people with normal chest radiograph findings was tested and found to have tuberculosis. This finding demonstrates how chest radiography is susceptible to human bias since abnormalities may be missed or misinterpreted as unrelated to tuberculosis despite subsequent bacteriologic confirmation. A recent study from South Africa found a large proportion of asymptomatic tuberculosis, where chest radiography only was able to detect 60% of tuberculosis overall, outperforming symptom screening.^38^ One potential reason may have been heterogeneity in chest radiograph interpretation, especially in health-care facilities that do not primarily manage tuberculosis.^39^ The 2021 *WHO consolidated guidelines on tuberculosis: module 2: screening: systematic screening for tuberculosis disease* recommends the use of artificial intelligence (AI) software for interpreting chest radiographs in tuberculosis screening and triage;^40^ studies in Viet Nam and elsewhere have successfully demonstrated that AI can complement radiographer interpretation and help to reduce false-negative chest radiograph results.^31^^,^^41^ Recent studies have also observed high proportions of tuberculosis diagnoses from repeat testing during follow-up among individuals with negative bacteriological results but abnormal chest radiograph findings, which could further enhance positivity.^42^^,^^43^

Our study demonstrated the substantial utility of chest radiography beyond improving facility-based diagnostic yield through detection of asymptomatic tuberculosis. When used as a first evaluation tool in a diagnostic algorithm among symptomatic individuals, chest radiography is valuable for improving pre-test probability and positive predictive value, while also ruling out tuberculosis and reducing molecular testing as demonstrated in large systematic reviews.^44^ We observed that chest radiograph screening had a slightly higher sensitivity than symptom screening for tuberculosis detection yet required a fraction of the diagnostic tests, reducing costs. Based on reported health-care system costs for tuberculosis care and commercial prices in Viet Nam, unit costs per molecular test and chest radiography were around 29 United States dollars (US$) and US$ 2.60, respectively, in 2023. The cost of screening 1 084 571 symptomatic participants with and without cough by chest radiography was about US$ 2.8 million. However, the cost of providing all 933 264 symptomatic individuals who had a normal chest radiograph with molecular testing would be US$ 27.1 million, implying theoretical savings of US$ 24.3 million.^45^ Further economic analysis to precisely quantify the benefits of chest radiography in facility-based screening is needed.

Despite its utility, there are well-known risks and potential harms in the use of chest radiography. A key risk is the radiation exposure and the accompanying stochastic effects and associated carcinogenicity for patients and radiographers. This risk is elevated in key populations such as children and pregnant women.^46^ Despite the “as low as reasonably achievable” principle and similar radioprotective protocols comprising fundamental tenets of radiography worldwide, their application may be variable and impaired by the overuse of medical imaging, particularly in low-resource settings of high tuberculosis burden.^47^ Concordantly, many facilities with high patient volumes included in our study were inherently biased towards over-reading chest radiographs and over-prescribing diagnostic tests, as is usual practice in Viet Nam. Furthermore, past studies have measured high heterogeneity and inter-reader variability in training and routine clinical practice of chest radiograph interpretation for tuberculosis abnormalities in Viet Nam, particularly among less-experienced radiologists. This issue may lead to both under- and overdiagnosis, and risk diagnostic delays, unwarranted anxiety and suboptimal use of resources.^48^ Nevertheless, a recent review concluded that, when appropriately justified, the benefits of radiography outweigh the low associated risks.^49^

Our study has several limitations. First, the retrospective nature of our study meant that only a descriptive analysis was possible. As such, causative factors that may explain why a participant would seek health care, the proportions of participants from specific hospital departments or vulnerable subgroups, the exact proportion of first- and second-generation molecular tests used, or other reasons for variable diagnostic accuracy in stratified analyses were unavailable. Second, we could not quantitatively characterize why participants with a normal chest radiograph, especially those without symptoms, received a molecular test. However, these participants represented a negligible portion of the total study population and of the asymptomatic cohort. We made the analytical choice to include these individuals, as opposed to presenting an approach based purely on chest radiograph results, to reflect the real-world conditions under which the algorithm was deployed. Third, as mentioned previously, a limitation of our study was the heterogeneity in identifying tuberculosis-related abnormalities on chest radiograph. These limitations may have affected our estimates of diagnostic yield and numbers needed to screen. Fourth, our study was based in a single country, and represented only adults presenting at general and specialty health-care facilities, limiting generalizability with respect to setting and target population. 

The strengths of our study are that we documented the results of chest radiograph screening for intensified case finding at a large scale that reflects real-world complexities, such as testing in participants with lower pre-test probabilities and despite normal chest radiograph results. The study also covered a wide range of health-care facilities at different levels of the health-care system, including the private sector.

We have demonstrated that chest radiograph screening can enable the diagnosis of tuberculosis in asymptomatic patients, and reduce the number of expensive molecular diagnostic tests used. Facilities with radiographic capacity should consider using chest radiograph as a screening tool for tuberculosis. Given the scale of intensified case finding and the potential of chest radiograph to save diagnostic costs, future research should include an analysis of comparative cost and cost–effectiveness for active and intensified case finding, as well as modelling these at a population level to understand the total impact of chest radiograph in both community and health-care facility settings.
